# The Relationships Between Stress, Mental Disorders, and Epigenetic Regulation of BDNF

**DOI:** 10.3390/ijms21041375

**Published:** 2020-02-18

**Authors:** Zhuang Miao, Yan Wang, Zhongsheng Sun

**Affiliations:** 1 Institute of Genomic Medicine, Wenzhou Medical University, Wenzhou 325000, China; miaomao357753@163.com; 2 Beijing Institutes of Life Science, Chinese Academy of Sciences, Beijing 100101, China; wangy@biols.ac.cn; 3 Department of Physiology, University of Toronto, Toronto, ON M5S 1A8, Canada; 4 School of Life Sciences, University of the Chinese Academy of Sciences, Beijing 100049, China

**Keywords:** brain-derived neurotrophic factor, mental disorders, stress, epigenetic regulation, sex differences

## Abstract

Brain-derived neurotrophic factor (BDNF), a critical member of the neurotrophic family, plays an important role in multiple stress-related mental disorders. Although alterations in BDNF in multiple brain regions of individuals experiencing stress have been demonstrated in previous studies, it appears that a set of elements are involved in the complex regulation. In this review, we summarize the specific brain regions with altered BDNF expression during stress exposure. How various environmental factors, including both physical and psychological stress, affect the expression of BDNF in specific brain regions are further summarized. Moreover, epigenetic regulation of BDNF, including DNA methylation, histone modification, and noncoding RNA, in response to diverse types of stress, as well as sex differences in the sensitivity of BDNF to the stress response, is also summarized. Clarification of the underlying role of BDNF in the stress process will promote our understanding of the pathology of stress-linked mental disorders and provide a potent target for the future treatment of stress-related illness.

## 1. Introduction

When a person suffers from stress, there may be severe damage to multiple organs and systems. If the central nervous system (CNS) is affected, individuals may develop mental disorders, such as anxiety and depression, which will be detrimental to their lives [1,2]. Brain-derived neurotrophic factor (BDNF), a well-known neurotrophic factor, is broadly expressed among the various brain areas [3]. BDNF plays an important role in the maintenance and survival of neurons, and it regulates neurogenesis in specific brain regions, including the subventricular zone (SVZ) and dentate gyrus (DG) [3,4,5]. Before conversion to mature BDNF (mBDNF), the precursor of neurotrophin is first synthesized, which is known as proBDNF [5,6]. There are two receptors for BDNF, namely, tropomyosin receptor kinase B (TrkB) and p75 neurotrophin receptor (p75^NTR^), which respectively exhibit high affinity to mBDNF or proBDNF and perform distinct functions [6,7]. As critical elements to modulate cell activities, some metal ions, such as Cu^2+^ and Zn^2+^, have been reported to activate BDNF signaling and facilitate the conversion from proBDNF to mBDNF [8,9,10]. In this review, we mainly discuss the alterations of mBDNF levels in response to diverse stresses. BDNF is secreted by various cells, including neurons, and can pass the blood–brain barrier [11]. A previous study also demonstrated the positive relationship of BDNF levels with the CNS and the peripheral system [12]. Moreover, as the BDNF protein in the brain has been implicated in the development of mental disorders [13], several research works have also proposed that peripheral BDNF can be considered as a potential marker of these diseases in humans [14,15]. To investigate the underlying relationship between stress and mental illness, many stress models have been established, although the intricacies of these models are not identical. The role of BDNF expression in stress-induced disorders has been examined but still remains unclear. In this review, we summarize current research regarding how different types of stressors influence BDNF expression in specific brain regions and their relationship with various mental illnesses. Epigenetic regulation of BDNF in stress-associated anxiety and depression is further discussed. In view of the multiple transcripts of BDNF and their particular functions, we also summarize specific alteration of the primary *Bdnf* isoforms in response to stress. Furthermore, we illustrate that other critical factors may influence BDNF in stressed individuals, such as sex, stress patterns, and duration of stress.

## 2. BDNF Expression and Mood-Related Brain Regions

BDNF, a member of the neurotrophin family, plays a critical role in the occurrence of mental illnesses, such as depression and anxiety [16,17]. Previous research has also demonstrated the relationship between stress and expression of BDNF in many critical brain regions [18]. Stressors have been shown to influence brain function through two distinct systems: the stress-related system, which mainly contains the hypothalamus–pituitary–adrenocortical (HPA) axis and the hippocampus, and the reward-associated system, which mainly contains the ventral tegmental area (VTA)–nucleus accumbens (NAc) pathway. BDNF may exert special influences on these two systems [13].

In the hippocampus, the primary brain area of the stress system, BDNF expression is significantly affected by extra stress. For instance, the social defeat procedure, which induces long-lasting depression-like behaviors in rodents, can downregulate the expression of *Bdnf* transcripts in the hippocampus, and this reduction can be reversed by chronic treatment with antidepressants [19]. Pollak and colleagues utilized the learned safety process to prevent the influences of stress in mice, and this inhibitory effect relied on the hippocampus neurogenesis [20]. Elevated BDNF expression in the hippocampus was also detected in this prevention, which suggests a negative relationship between stress-related depression and hippocampal BDNF levels. Stress-associated anxiety-like phenotypes, which may coexist with depression-like phenotypes, were also demonstrated to be related with the reduction of BDNF in the hippocampus [21,22]. Although there are different phenotypes in anxiety and depression, antidepressants are reported to treat a set of anxiety-related phenotypes [23], which indicates the existence of shared mechanisms in these two disorders, which probably relies on BDNF. The amygdala is another critical brain region that mainly regulates emotional behaviors, the abnormality of which is involved in stress-related mental disorders, especially anxiety [24,25]. Previous studies have shown that stress exposure can lead to anxiety-associated phenotypes in rodents, which is consistent with the upregulation of BDNF in the amygdala [26,27].

In the reward system of rodents, levels of BDNF in the VTA and NAc control resistance and susceptibility to stress stimulation [28,29], which can indirectly influence the induction of adverse behaviors. The majority of research on the medial prefrontal cortex (mPFC) has shown a correlation between decreased BDNF and anxiety- and depression-like behaviors, while the VTA–PFC projection implies that the upregulation of BDNF in the mPFC may be responsible for other phenotypes related to reward. Although anxious and depressive behaviors are usually comorbid [30], there are distinct characteristics in these two symptoms. Sonia and colleagues found that individuals with anxiety tend to avoid threat, whereas depression may be mostly identified as decreased pursuit of reward [31]. Considering the different changes in BDNF in stress-related (decreased BDNF in hippocampus) and reward-related systems (increased BDNF in VTA and NAc), stress-induced anxiety and depression may be associated with the degree of activation of different pathways. Another study also demonstrated that both depressive and anxiety-like behaviors were related with the elevation of proBDNF in the hippocampus, whereas dendritic arborization and synaptophysin in neurons were differently regulated in these two phenotypes [32]. This research suggests the importance of morphological plasticity and supports the “neurotrophic hypothesis” [33], which postulates that stress-induced adverse phenotypes may be due to the loss of cells lacking neurotrophic maintenance.

## 3. The Effects of Different Stress-Related Behavioral Paradigms on BDNF Expression

Multiple animal models have been utilized to mimic the stress situation. The restraint stress procedure represents the most commonly used physical stressor [34,35], whereas the widely used psychological stressor procedures include elevated platforms, predator odors, and maternal separation (MS). Some models can induce both physical and psychological stress, such as repeated exposure to the social defeat procedure [36]. Previous reports have described the possible differential consequences between physical stress and psychological stress on animal behaviors [37,38]. As a critical neurotrophin that regulates synaptic plasticity, neurogenesis, and the outcomes of behaviors, BDNF has been studied in many of these stress-related behavioral paradigms.

### 3.1. Restraint Stress

Restraint stress is one of the most widely used stress paradigm in rodents. For acute restraint stress (ARS), some studies have reported decreased BDNF mRNA and protein levels in the prefrontal cortex and hippocampus but increased BDNF expression in the amygdala of rodents [26,39,40,41,42]. However, other studies have also shown the opposite trend in Bdnf mRNA levels in these brain areas following acute stress [43,44]. Inconsistent alterations in Bdnf mRNA levels in acute restraint stress may depend on the time course of the stress [45,46]. It has been reported that Bdnf mRNA levels are upregulated at the early phase and then downregulated later in the hippocampus. Similar to ARS, the majority of chronic restraint stress (CRS) has been found to reduce BDNF levels in the hippocampus and prefrontal cortex but upregulate its expression in the amygdala of rodents [26,47,48]. In addition, Yeh Che-Ming and colleagues stated that prenatal CRS can downregulate mBDNF levels in the hippocampus of rats by preventing the conversion from proBDNF to mBDNF, thus upregulating the levels of proBDNF [49].

Moreover, the difference in BDNF alterations in target brain regions induced by acute and chronic restraint stress have been demonstrated. For example, Nair and colleagues stated that although ARS decreases all Bdnf transcripts in the hippocampus, the CRS can increase some variants in the same region [50]. In addition, although both acute and chronic restraint stress can result in the reduction of BDNF expression in the hippocampus, chronic restraint stress shows a less robust effect [51].

In summary, restraint stress, no matter acute or chronic, can lead to abnormal mood-related behavior in rodents, which is associated with altered BDNF levels in specific brain regions. The effects of this physical stress may depend on the duration of the stress, the species, and the period in which the stress occurs. Moreover, the time-dependent expression of BDNF and different Bdnf isoforms may also play an important role in regulating BDNF levels after stress.

### 3.2. Foot Shock Stress

Foot shock stress is commonly used in the fear conditioning test or learned helplessness test to study the mechanisms of learning and memory or other abnormal behaviors in rodents. Altered BDNF levels in the hippocampus of rodents have been proven to play an important role in stress-induced behaviors [52]. The results of functional magnetic resonance imaging (fMRI) have shown that application of foot shock combined with conditioned light stimulation can activate fear circuitry in some brain regions, such as the amygdala and granular insular in control rats but not in BDNF heterozygous rats [53]. The intensity of electric stimulation appears to be important for BDNF regulation in this stress model. For example, after increasing the intensity of foot shock in a re-exposure process, BDNF expression was reduced more dramatically in the DG [54]. In addition, BDNF levels in the hippocampus were increased more in female rats suffering low-intensity stimulation than in those suffering high-intensity stimulation [55]. Notably, although acute and chronic shock stress could reduce and elevate BDNF in the DG and mPFC of female rats, respectively, a similar phenomenon was not observed in male rats [56], suggesting the sex-specific effect of foot shock stress.

Previous studies have also demonstrated the distinct influences on brain regions between the single application of foot shock stimulation and foot shock combined with other conditioning factors. For example, Bdnf mRNA expression was not changed by a single application of foot shock; however, after the addition of predator odor exposure, it was significantly elevated in the olfactory bulb, piriform cortex, and amygdala [57]. The various results in response to additional emotional elements under this physical stress emphasize the necessity to distinguish between physical and psychological stressors in further research.

### 3.3. Predator Odor Stress and Elevated Platform Stress

Pregnant mice exposed to predator odors can develop several abnormal behaviors, including impaired maternal care. A large amount of research has demonstrated that exposure to predator odors can influence the expression of BDNF in multiple brain regions in mice [58,59]. Moreover, a previous study of mice showed that levels of Bdnf transcripts in the hippocampus were simultaneously decreased by this prenatal stress exposure but not in the amygdala of the female offspring [60], suggesting a tissue-specific effect.

Immunization treatment with a specific antigen in the CNS, which affected the T-cell trafficking, could restore BDNF levels in the hippocampus of mice and was found to be related to the resilience of predator stress [61]. Another study also found that immune-deficient Rag2(-/-) mice exhibited reduced startle response to noise after predator odor stimulation, which was associated with an elevated level of mature BDNF (mBDNF) in the hippocampus. However, this resilience did not arise through fear conditioning and learned helplessness [62]. These findings may indicate important interactions among immunity status, neurotrophin, and specific stressors.

In the elevated platform stress paradigm, levels of BDNF protein are downregulated in the hippocampus and frontal cortex of rats [63,64]. Interestingly, another work has shown that elevated platform stimulation can transfer short-term memories to long-term memories, which depends on the upregulation of BDNF in the hippocampus [65]. The contradictory results of psychological stressors on BDNF expression and related phenotypes indicate the complexity of stress-related behaviors.

### 3.4. Maternal Separation

The absence of maternal care can induce behavioral abnormalities; therefore, it is usually utilized in stress models, such as maternal separation or deprivation, to exert early-life stress in rodents. MS is applied during the first three weeks postnatally, known as the juvenile period. However, MS can result in long-lasting effects, which can be maintained into the adult period. The alteration of BDNF levels induced by MS shows an age- and tissue-dependent effect to a large extent. For example, after suffering MS, BDNF expression of both CA1 and DG were increased at the adolescent age in rats. Notably, only the upregulation of BDNF expression in the DG region was maintained into the adult period [66]. Furthermore, BDNF levels in the mPFC were decreased when the stressed rats reached adulthood. Lee and colleagues also reported that rats experiencing MS exhibited enhanced BDNF levels at the juvenile stage, then recovered to baseline levels during adolescence in the mPFC, with neurotrophin gradually decreasing in the adult phase [67]. Although these time-dependent fluctuations in BDNF expression also appear in animal models of restraint stress, most rodents are exposed to this physical stress in the adult period, and the influence of stressors may be transient. MS stress occurring in the early phase of life can dramatically disrupt brain development and hence induce long-term changes, which may be the reason for the age-dependent effect of MS. Moreover, some studies report increased BDNF and decreased BDNF response to MS in the hippocampus and mPFC of rodents, respectively [68,69]; however, others do not [70,71]. The contradictory results may be related to the age of the animal as well as the duration of stress.

Compared to the restraint physical stressor and the other two psychological stressors (elevated platform and predator odor), the MS stressor exhibits more extensive influence on the brain, which is speculated to be associated with the social factor in this stress model. For example, besides the hippocampus and prefrontal cortex, which are susceptible to stress, MS has been reported to affect BDNF expression in the amygdala [27,72], nucleus accumbens [73], VTA [74], striatum [74,75], cerebellum [76], and hypothalamus [77,78] of rodents. In humans, a previous study showed a positive association between serum BDNF levels and maternal overprotection in the Val66Met mutant [79]. Grassi-Oliveira and colleagues found an association between decreased levels of plasma BDNF and childhood-neglect-induced depression and memory impairment [80], which lends support to the possible wide impacts of the social psychological stressor and is worthy of further study.

### 3.5. Post-Traumatic Stress Disorder (PTSD) and Witnessing Stress

Post-traumatic stress disorder is a severe symptom triggered by experiencing adverse events, such as abuse, war, terrorist attack, and accidents during one’s life [81]. BDNF has been reported to be correlated with the development of PTSD [82,83]. Recently, Warren and colleagues utilized the native empathy of rodents to design a novel type of social psychological stress model, in which male mice were allowed to witness defeat events and displayed anxiety- and depression-like phenotypes [84]. Based on this model, our group used social witnessing stress to stimulate female mice and observed anxiety-like behaviors, which were correlated with decreased BDNF in the hippocampus and mPFC and increased BDNF in the amygdala [85]. Similar to this study, other studies have also shown that stress may cause different influences on the BDNF levels in different brain regions [26,66,68]. This may be due to the fact that there are close interactions among brain regions, and the neural activities in one area may regulate those in other regions. For instance, through pharmacological treatment with d-amphetamine and glucocorticoid receptor agonists, McGaugh and colleagues have proven that the input from amygdala can significantly modulate the formation of hippocampal memory [86,87]. In addition, although both the hippocampus and amygdala can respond to stress and further affect the activities of the HPA axis through the GABAergic neurons, the feedback of these two limbic regions to the HPA axis are opposite [88], which may explain the different alterations of BDNF in specific areas following the same stress stimulation. Considering that the MS stressor can only be applied during early life, this witnessing emotional stress model will be a potent tool for exploring the mechanisms of complicated interactions between social-stress-induced symptoms, such as PTSD, and neurotrophins throughout the whole life.

### 3.6. Repeated Social Defeat Stress

The repeated social defeat stress model is widely used in rodents. This type of stress has been proven to cause a range of mental disorders, including anxiety-like and depression-like phenotypes [28,36]. The social defeat process is divided into two parts: the direct defeat period, which leads to physical harm to experimental rodents, and the following psychological threat period, which avoids bodily contact between the attacker and the stressed animal. Similar to the MS stressor, social defeat stress can influence multiple brain regions and lead to adverse behaviors in rodents. For example, Nestler and colleagues found that social defeat stimulation can induce social avoidance and anxiety-like and depression-like behaviors in mice and that BDNF levels within the NAc are correlated with susceptibility to this stress [28,89]. In the same research, by examining human postmortem brain samples, they also found a positive correlation between BDNF levels in the NAc and depression, highlighting the clinical relevance of this social defeat model to neurotrophin and mental disorders.

The VTA region, another essential brain area in the reward circuit, was also reported to be affected by the defeat stressor and associated with changed BDNF levels in rodents [90,91]. Along with the organization of dopamine neurons in mesolimbic dopamine pathways, the VTA also projects to the mPFC and amygdala, besides the NAc, through these pathways [92]. Nikulina and colleagues found that BDNF levels were elevated in these areas after social defeat stimulation [93], which may indicate the synergetic role of BDNF in dopamine pathways and associated deleterious behaviors. Another research has verified that the extra-activation of the VTA–NAc pathway during the social defeat procedure can exaggerate the abnormal phenotypes induced by this stress, whereas these behaviors are reversed to normal conditions by blocking the BDNF/TrkB pathway in the NAc or VTA [29,94]. However, this work also emphasizes that the recovery of behaviors relies only on BDNF signaling and not the dopamine pathway, suggesting the importance of BDNF/TrkB signaling in the VTA–NAc reward circuit of behaviors related to social defeat stress.

In addition to the classical VTA–NAc pathway, the social defeat stressor has also been demonstrated to affect the expression of BDNF in various other brain regions. For example, rats with low-novelty-seeking behavior are resistant to induction of depression-like behaviors after social defeat stress, which is correlated with the upregulation of BDNF in the hippocampus [95]. In contrast, decreased BDNF in the hippocampus is implied to participate in social-defeat-induced anxiety-like symptoms [21,96]. For the amygdala, BDNF levels have been reported to be elevated after the social defeat paradigm, which is associated with social avoidance and submission learning in mice [97,98]. For the mPFC, although some research works have stated that social defeat stress upregulates BDNF expression [99], there is still a subset of studies that did not observe significant alterations [21,100] or proposed the opposite, downregulation effect on BDNF expression [101,102]. In addition, several studies have also suggested the time-dependent effect of BDNF expression in the mPFC of rodents after they experienced defeat stress [90,103], which may explain the different observations mentioned above.

Due to the individual differences in attacking intensity, social defeat stress cannot be accurately quantified. Moreover, female rodents are generally without aggressive motivation; therefore, this defeat procedure is usually restricted to males. Although in a recent study, researchers successfully established a new social defeat model in female mice by applying the male odorants on them to increase their aggressive behaviors, the related study on females is lacking [104]. In view of these obvious disadvantages, which may be the main reasons for the contradicting results underlying this stress model, more effort should be made in modifying uncontrollable pain and sex differences.

The influence of different stressors on BDNF changes in various stress models is summarized in Table 1.

## 4. Epigenetics, Stress, and BDNF

The Bdnf gene possesses multiple exons, each of which has a specific promoter allowing the formation of various transcripts [106,107,108]. Based on different splicing patterns, the various Bdnf transcripts are distributed to many brain regions. Different stressors can regulate the levels of different Bdnf transcripts in specific areas. For example, maternal predator odor exposure can lead to downregulation of Bdnf exon IV in the hippocampus of female rat offspring [109]. Another study of prenatal stress models demonstrated the positive relationship between Bdnf exon IV and IX levels in the frontal cortex and hippocampus of mice and social interaction activities [110]. Neeley and colleagues also stated that prenatal stress can increase hippocampal Bdnf exon VI expression in rats, although exhibiting differences across strains [111]. After exposure to stress, the expression of Bdnf transcripts was elevated in the amygdala of rodents [112,113]. In contrast, elevated Bdnf exon IV expression was observed in the hippocampus of rats, which exhibited resistance to acute unavoidable stress [114]. In the cortex of mice with increased Bdnf exon I and IV, electroconvulsive therapy can recover dendritic spine impairments caused by chronic stress [115].

Moreover, several research groups have reported that exposure to stressors may induce alterations of distinct Bdnf transcripts. For instance, context stimulation can upregulate Bdnf exon I and VI in the hippocampus of rats [116]. In addition, while acute immobilization stress downregulates all Bdnf transcripts, chronic stress upregulates Bdnf exon I and II in the hippocampus of mice [50]. In the same study, the modification of Bdnf variants exhibited time-dependent effects after stress stimulation. Specifically, elevation of Bdnf II at postnatal day 14 and Bdnf exon IV/V at postnatal day 21 was detected. This time-dependent impact on Bdnf variants was also described in another independent study [112]. Some studies have reported that, after treatment with pilocarpine, a model of epileptogenesis, Bdnf II and VI isoforms accumulated at the dendrites of rat hippocampal neurons, whereas Bdnf I and IV were restricted to the somatic regions [117]. This specific spatial distribution of Bdnf isoforms may be associated with the different phenotypes induced by extra stressors.

Epigenetic modulation, which consists of DNA methylation [118], histone modification [119], and regulation of noncoding RNAs [120], plays a pivotal role in the differential expression of Bdnf variants and impacts specific mental disorders due to its distinct functions. We will now highlight recent studies of these epigenetic mechanisms and their relationship with the stress-related changes in Bdnf variants.

### 4.1. DNA Methylation and BDNF

DNA methylation, one of the main epigenetic regulators, can regulate the expression of target genes through their promoter regions [121]. Normally, Cytosine—phosphate—Guanine (CpG) sites at the promoter regions are unmethylated, whereas the transcription of target genes is repressed once methylation occurs. The DNA methyltransferases (DNMTs) transfer a methyl group to the fifth position of cytosine and maintain the context of CpG sites [122]. The methyl-CpG-binding proteins (MeCPs) recognize the target methylated CpG regions and further recruit specific complexes to suppress gene transcription [123,124]. Ten-eleven translocation enzymes (TET) can transfer 5-methylcytosine (5mC) to 5-hydroxylmethylcytosine (5hmC) and demethylate DNA [125,126].

BDNF, including its multiple transcripts, has been shown to be regulated by DNA methylation in many studies. For example, stress can induce abnormal behaviors in rats, which is accompanied by increased DNA methylation at the Bdnf exon IV promoter and thus decreased BDNF expression in the hippocampus [127,128]. Stress can also influence the expression of specific Bdnf isoforms through DNA methylation in other brain regions. For example, male rats, but not females, suffering from prenatal stress exhibited elevated methylation levels at the Bdnf exon IV promoter in the mPFC [129]. In addition to the primary Bdnf exon IV, other isoforms of Bdnf mRNA were also influenced by stress-related DNA methylation. Notably, altered DNA methylation at the BDNF promoters were found in blood samples of stressed humans with depressive symptoms [130,131].

Compared to the effect on the specific promoters of each transcript, stressors can also modulate multiple promoter regions of different Bdnf variants. For instance, prenatal restraint stress decreased the expression of Bdnf transcripts (III, IV, VI, and IX) in the frontal cortex and hippocampus of adult rats [110]. Male rats experiencing caregiver maltreatment, in which the dams were exposed to insufficient nesting materials and a new environment, developed increased methylation levels at the promoter of Bdnf exon I in the hippocampus and decreased Bdnf exon IV in the amygdala [132]. Relying on the distinct functions of DNMTs, MeCPs, and TETs, the balance of gene expression is maintained through regulation of their DNA methylation, and stressors have been reported to interrupt this balance. Kumar and colleagues demonstrated that hypobaric hypoxia can reduce BDNF protein in the hippocampus of rats, which is related to increased DNMT1, DNMT3b, and MeCP2 expression [133]. MS stress can increase the levels of Dnmt3a, MeCP2, and Tet3 in the amygdala of adolescent or adult rats, which is associated with decreased BDNF expression [134]. The alteration of Bdnf transcripts by prenatal restraint stress is also positively related to increased DNMT1 and TET1 and an enrichment of 5mC and 5hmC levels at the specific promoter regions of Bdnf variants in mice [110].

Notably, DNA methylation at the Bdnf promoters may not be a crucial factor in some stress-related behaviors. For example, although reduced methylation at the CpG sites of Bdnf promoters was found in the blood samples of veterans who had PTSD symptoms, the levels of BDNF mRNA did not change significantly [135]. In addition, Miao and colleagues reported that mice suffering from psychological stress showed downregulated BDNF levels in the hippocampus, which is not associated with DNA methylation at the promoter regions [85].

### 4.2. Histone Modification and BDNF

Aside from DNA methylation, chromosomal histone modification, which includes methylation, acetylation, and phosphorylation, is another common epigenetic regulation for regulating the expression of genes [136]. Histone methylation and demethylation occur on multiple residues and play an important role in biological functions such as stress response, DNA repair, and differentiation [137]. One study showed that rats suffering MS stress showed reduced histone methylation levels in the promoter of Bdnf exon IV with increased BDNF expression in the hippocampus [138]. Fasting stress in chicks caused increased H3K27 methylation at the promoter of Bdnf, which is correlated with the repression of BDNF expression in the hypothalamus [139]. Histone acetylation at N-terminal lysine is regulated by histone acetyltransferases (HATs) and deacetylase (HDACs) [140]. Chronic restraint stress results in the reduction of Bdnf exon IV, H3 acetylation in the related promoter in the hippocampus of rats, and the elevation of HDAC5 [141]. Moreover, in rats, single immobilization stress can downregulate Bdnf exon I and IV, which is positively related with the decreased histone H3 acetylation at these promoters [142]. Altered acetylation levels of specific histone residues, such as H3K9, H3K14, and H4K12, at the promoters of Bdnf isoforms were also detected in stress models [143,144,145]. In contrast, HDAC-induced histone deacetylation was found in the stress-related downregulation of BDNF in some brain regions of rats [146].

Histone modifications seemingly occur at different Bdnf promoter regions to regulate stress-related behaviors. Nestler and colleagues found that social defeat stress can induce depression-like behaviors and decrease Bdnf III and IV in the hippocampus of mice; this downregulation of Bdnf transcripts is accompanied by increased histone methylation of related promoters [19]. Chronic treatment with antidepressants can reverse the reduction of BDNF and elevate histone acetylation at the promoter regions of these Bdnf isoforms, which is associated with decreased HDAC5. MS stress triggers cognitive deficits in Balb/c mice, but not C57BL/6J, and this phenotype is correlated with HDAC1 levels at the promoter of Bdnf variant III [147]. These results suggest a strain-specific influence on histone modification in stress-associated behaviors. Sex was also shown to affect histone modification of the Bdnf promoter after stress exposure. For example, female rats, but not males, had downregulated H3K9/14 acetylation at the promoter regions of Bdnf IV in the mPFC after experiencing MS [148].

### 4.3. Noncoding RNA Regulation and BDNF

miRNAs are a series of small noncoding RNAs, which post-transcriptionally modulate the mRNA of target genes by binding to the 3’-untranslated regions (3’UTR) [149]. Numerous miRNAs have been found to play a critical role in regulating BDNF expression following stress stimulation. For example, neonatal isolation stress can lead to upregulation of miR-124 in the hippocampus of adult rats, accompanied by anxiety-like behaviors, social avoidance, and decreased BDNF levels [150]. In the social defeat stress model, a negative correlation between miR-30a and BDNF was found in the hippocampus of mice, which also exhibited similar impaired social interaction and anxiety-like phenotypes after stress exposure [21]. Moreover, following chronic unpredictable mild stress, rats showed depression-like behaviors and decreased BDNF and increased miR-182 in the hippocampus [151]. On the contrary, the upregulation of miR-182 can reduce the target BDNF levels and exacerbate depression-like phenotypes.

Other microRNAs, such as miR-10b and miR-16, which can directly regulate BDNF levels, have been reported to participate in stress-induced anxiety- and depression-like behaviors of rats [152,153]. In addition, another study demonstrated that social witnessing stress can affect miR-206 levels and negatively regulate BDNF in the hippocampus, mPFC, and amygdala of mice [85], which suggests the wide range of regulatory effects of microRNA in the stress process.

In humans, patients with major depressive disorder (MDD) showed reduced levels of BDNF transcripts in the prefrontal cortex. A similar phenomenon was also detected in C57BL/6J mice that experienced chronic stress [154]. By utilizing short hairpin RNA (ShRNA) to target Bdnf long-3’UTR, the expression of Bdnf transcripts can be selectively suppressed in the mPFC of mice and cultured neurons, which can lead to stress-associated behaviors and MDD-like gene alterations.

The mainly epigenetic regulation on stress-related BDNF alterations is summarized in Table 2.

## 5. Sex Differences in Stress-Associated BDNF Alterations

Stress has been demonstrated to induce sex-specific effects [155,156], although the reasons remain unclear. As BDNF has been shown in some studies to play an important role in stress susceptibility [157], it is reasonable to consider BDNF a candidate target for stress-related sex differences. Previous preclinical and clinical research has supported the finding that females with abnormal phenotypes and BDNF levels in specific brain areas are more vulnerable to stress than males. For instance, female mice with BDNF deletions in the forebrain or hippocampus are more sensitive to chronic unpredictable stress and exhibit depression-like behaviors; on the contrary, loss of BDNF in the same brain regions does not increase stress susceptibility in male mice [158,159]. MS stress can increase sensitivity to neuropathic pain on inducing depression-like behaviors only in female mice, but not in males, which is associated with differential BDNF expression in the hippocampus and striatum between the two sexes [160]. In rats, exposure to prenatal stress only selectively reduces the expression of BDNF in the hippocampus in females. This sex-specific difference also appears when the adult suffers acute stress and may rely on epigenetic mechanisms [43]. In another research on rats, Hill and colleagues reported that female exposure to MS plus corticosterone treatment exhibited anhedonia, which was coupled with decreased mBDNF in the ventral hippocampus [161]. Interestingly, the males, which suffered the same stress, showed impaired short-term memory, which was associated with increased Bdnf expression but decreased mBDNF in the dorsal hippocampus.

Except for the female-specific effects caused by stress, other studies have also shown the existence of stress-induced male-specific effects on BDNF. For example, male Bdnf heterozygous mice treated with stress hormone were reported to show impaired memory [162]. Preterm infants are considered to suffer pain stress in early life. It has been reported that preterm boys, but not girls, with the BDNF Val66Met variant, which reduces BDNF secretion, are vulnerable to this kind of stress and show altered cognitive performance [163]. There are several explanations for these stress-related sex differences: (I) Stress can modify BDNF expression in specific brain areas based on different genders. (II) The region-specific alteration of BDNF may lead to distinct phenotypes in both sexes. As mentioned above, MS stress results in a deficit of memory and anhedonia in male and female rats, respectively, with both hippocampi showing the absence of BDNF [161]. (III) the age of individuals experiencing stress may play a critical role in this sex-difference. As demonstrated in the work of Maarten and colleagues, male Bdnf heterozygous mice induced with corticosterone stress developed impaired memory at the age of 11 weeks, but this behavior disappeared at 15 weeks [164]. (IV) The intensity of the stressor may contribute to this sex difference. For instance, previous research has shown that, compared to male rats, females are more sensitive to low-intensity foot shock stress, which can improve the learning process in the water maze test, and this performance is coupled with upregulated BDNF levels in the hippocampus [55].

We have summarized the stress-induced sex-specific effects on BDNF in Table 3. These findings indicate that stress can influence specific brain regions, leading to altered BDNF levels correlating with mental disorders, in a sex-dependent manner. Notably, most studies examining the male-specific effect in mice and humans were carried on heterozygous individuals [162,163,164,165], which means the abnormal expression of BDNF is not confined to particular brain regions. The mechanisms underlying this sex-specific effect on BDNF may be associated with various elements, including brain regions, age, types of stressors, and species, all of which should be further clarified precisely.

## 6. Conclusions

In this review, we have summarized the effect of stress on BDNF and its relationship with various mental disorders. The alterations of BDNF after stress exposure are regulated by DNA methylation, histone modification, and small noncoding RNA, which are dependent on specific brain regions, specific isoforms, sexes, as well as the patterns, duration, and period of the stress, suggesting the essential and complex involvement of BDNF in mood regulation. Distinguishing the effects of these factors is necessary for clarification of the relationship between stress and BDNF, which will further facilitate the clinical treatment of stress-induced mental illness.

## Figures and Tables

**Table 1:** Summary of the effects of distinct stressors on brain-derived neurotrophic factor (BDNF).

Stress Patterns	Species	Stress Period	Phenotypes	BDNF Alterations	Reference
Acute restraint stress	Mouse	12 weeks	-	Hippocampus BDNF protein ↑	[41]
		8 weeks	Anxiety-/Depression-like	Hippocampus, PFC *Bdnf* mRNA ↓	[42]
	Rat	7–9 weeks	-	Hippocampus, *Bdnf* mRNA ↓	[40]
		Prenatal stress and 8 weeks	-	Hippocampus, PFC *Bdnf* mRNA ↑	[43,44]
		8 weeks	-	Amygdala, *Bdnf* mRNA ↑	[26]
Chronic restraint stress	Mouse	Adult 9–12 weeks	Depression-like Cognition impairment	Hippocampus BDNF ↓	[47,105]
	Rat	Prenatal stress	-	Hippocampus, proBDNF ↑ mBDNF ↓	[49]
		Adult	Depression-like	PFC, BDNF ↓	[48]
		8 weeks	-	Amygdala, *Bdnf* mRNA ↑	[26]
Predator odor	Mouse	Prenatal stress	-	Hippocampus, *Bdnf* mRNA ↓	[60]
Elevated platform	Rat	Adult	-	Frontal cortex, BDNF ↓	[63]
Maternal separation	Rat	Postnatal day (P) 1-21	Depression-like	Hippocampus, BDNF ↑ mPFC, BDNF ↓	[66,68]
		P1-14	-	Hippocampus, BDNF ↓	[70]
		P2-21	Anxiety-like	Amygdala, BDNF ↑	[27,72]
		P10	-	Hypothalamus, *Bdnf* mRNA ↓	[77]
		P2-14	-	VTA, BDNF ↑; Striatum, BDNF ↓	[74]
Social witnessing	Mouse	Gestation	Anxiety-like	Hippocampus, mPFC, BDNF ↓; Amygdala, BDNF ↑	[85]
Social defeat	Mouse	Adult	Anxiety-like Depression-like Social avoidance	NAc, BDNF ↑	[28]
		Adult	Depression-like	mPFC, BDNF ↓	[101]
	Rat	Adult	-	PFC, Amygdala; NAc, VTA, BDNF ↑	[90,93]
		Adult	Depression-like	mPFC, BDNF ↓	[102]

Note: PFC, prefrontal cortex; NAc, nucleus accumbens; VTA, ventral tegmental area; P, Postnatal day; -, not mentioned; ↑, the expression is elevated; ↓, the expression is decreased.

**Table 2:** Summary of epigenetic regulations on BDNF expression in stress models.

Epigenetic Regulation	Species	Stress Patterns	Stress Period	Phenotypes	BDNF Alterations	Ref
DNA methylation	Mouse	Restraint stress	Prenatal	Impaired social interaction	Hippocampus, FC *Bdnf* I-IV, VI, IX ↓	[110]
	Rat	Repeat FS or PS	Prenatal or adult	Anxiety-like	Hippocampus *Bdnf* IV ↓	[127,128]
		Unpredictable stress	Prenatal	-	mPFC, *Bdnf* IV ↓	[129]
	Human	Job stress	Adult	Depression	Blood, *BDNF* I promoter DNA methylation ↑	[130]
Histone Methylation	Mouse	Social defeat stress	9–11 weeks	Impaired social interaction	Hippocampus, H3K27me ↑ *Bdnf* III, IV ↓	[19]
	Rat	Maternal separation	P2-14	Impaired memory	Hippocampus Young adult: H3K9me ↑, *Bdnf* IV ↓, Middle-aged: H3K9me ↓, *Bdnf* IV ↑	[138]
	Chick	Fasting stress	Day 3 and 10	-	PVN, H3K27me ↑, BDNF↓	[139]
Histone Acetylation	Mouse	Restraint and light stress	Prenatal	Depression-/Anxiety-like	Hippocampus, H3K14ac ↓, BDNF↓	[144]
	Rat	Restraint stress	Adult	-	Hippocampus, H3 ↓ *Bdnf* I, IV ↓	[141,142]
		CUMS	5 weeks	Depression-like	Hippocampus H3K9ac ↓, BDNF ↓	[143]
		Social isolation and CUMS	Adult	Impaired memory	Hippocampus, H3K9ac, H4K12ac ↓, BDNF ↓	[145]
Noncoding RNA	Mouse	Social defeat	Adult	Anxiety-like	Hippocampus, miR-30a ↑ BDNF ↓	[21]
		Social witnessing	Adult	Anxiety-like	Hippocampus, mPFC, miR-206 ↑, BDNF ↓ Amygdala, miR-206 ↓ BDNF ↑	[85]
	Rat	Maternal separation or CUMS	P1-13 or adult	Impaired social interaction, Anxiety-/Depression-like	Hippocampus, miR-124a, miR-182, miR-10b, miR-16 ↑, BDNF ↓	[150,151,152,153]

Note: CUMS, chronic unpredicted mild stress; PS, psychosocial stress; FS, forced swimming; PVN, paraventricular nucleus; FC, frontal cortex; mPFC, medial prefrontal cortex P, Postnatal day; -, not mentioned; ↑, the expression is elevated; ↓, the expression is decreased.

**Table 3:** Summary of sex-specific modification on BDNF expression.

Sex	Species	Stress Patterns	Stress Period	Phenotypes	BDNF Alterations	Reference
Male	Mouse	CORT treatment	6–9 weeks	Impaired memory	*Bdnf* heterozygous mice	[162,164]
		Mild stress	4–6 months	Depression-like		[165]
	Rat	MS and CORT treatment	P2-14 8–10 weeks	Impaired memory	Dorsal hippocampus *Bdnf* transcripts ↑ mBDNF ↓	[161]
		Restraint stress	Prenatal	Anxiety-like	Hippocampus proBDNF, mBDNF ↑	[166]
		Maternal deprivation	P9-10	-	Hypothalamus, *Bdnf* mRNA ↓	[78]
	Human	Preterm	Infants	Impaired cognition	*BDNF* Val66Met variant	[163]
Female	Mouse	MS	2–3 weeks	Depression-like	PFC, BDNF ↓	[158,167]
		CUS	3–6 months	Depression-like	Hippocampus, mPFC BDNF ↓	[160]
		Restraint stress	Prenatal	-	Hippocampus proBDNF ↑, mBDNF ↓	[168]
	Rat	Restraint stress	Prenatal	-	mPFC, BDNF ↓	[43]
		MS and CORT treatment	P 2-14 and 8–10 weeks	Depression-like	Ventral hippocampus BDNF ↓	[161]
		Maltreatment	P1-7	-	Amygdala, *Bdnf* ↓	[169]
	Vole	Paternal deprivation	P1-21	-	Dentate gyrus, BDNF ↓	[170]

Note: CORT, corticosterone; CUS, chronic unpredictable stress; MS, maternal separation; PFC, prefrontal cortex; mPFC, medial prefrontal cortex P, Postnatal day; -, not mentioned; ↑, the expression is elevated; ↓, the expression is decreased.

## Abbreviations

BDNF	brain-derived neurotrophic factor
TrkB	tropomyosin receptor kinase B
p75^NTR^	p75 neurotrophin receptor
HPA	hypothalamus–pituitary–adrenocortical
CNS	central nervous system
PTSD	post-traumatic stress disorder
CORT	corticosterone
mPFC	medial prefrontal cortex
NAc	nucleus accumbens
VTA	ventral tegmental area
PVN	paraventricular nucleus
SVZ	subventricular zone
DG	dentate gyrus
MS	maternal separation
ARS	acute restraint stress
CRS	chronic restraint stress
fMRI	functional magnetic resonance imaging
DNMT	DNA methyltransferase
MeCP	methyl-CpG-binding protein
TET	the ten-eleven translocation enzyme
5mC	5-methylcytosine
5hmC	5-hydroxylmethylcytosine
HAT	histone acetyltransferase
HDAC	histone deacetylase
3’UTR	3’-untranslated regions
MDD	major depressive disorder
ShRNA	short hairpin RNA
